# Supplementary material for: Content-rich biological network constructed by mining PubMed abstracts
Source: BMC Bioinformatics. 2004 Oct 8;5:147. doi: 10.1186/1471-2105-5-147 (PMC528731; doi:10.1186/1471-2105-5-147)
Supplement: Additional File 2 — The original results of the above study (non-essential files are deleted to keep the file size under the limit set by BMC bioinformatics). [file 1471-2105-5-147-S2.bz2 › chilibotAdditionalFile2/dip20/623ID9103205E11009/html/GRPE_DNAK.html]

 


 **GRPE** and **DNAK** 
  
Found 298 abstracts in PubMed, retrieved 20.  
 

 What does Google say? 
 PDF only 
| .edu only 

---

**Interactive relationship** (e.g. stimulation, inhibition, etc)

**Neutral relationship**- This information is important for understanding the conformational changes  **GrpE**  undergoes on binding to  **DnaK** .  Ref: 12840016 J Biol Chem< MedlineTA>J Biol Chem,
- We show that the equilibrium dissociation constants for the interaction of  **GrpE**  with wtDnaK, lidless  **DnaK**  2 517, the ATPase domain 2 388, and the substrate binding fragment 393 507 are 64 16 nM, 4.0 1.5 nM, 35 10 nM, and 67 11 μM, respectively,.  Ref: 12885236 BiochemistryBiochemistry,
- Such results strongly suggest that the 33 residue flexible N terminal tail of  **GrpE**  binds in the substrate binding pocket of  **DnaK** .  Ref: 12885236 BiochemistryBiochemistry,
- Our results reveal a complex set of molecular interactions between  **DnaK**  and its cochaperone  **GrpE** .  Ref: 12885236 BiochemistryBiochemistry,
- The substrate binding properties of  **DnaK**  are controlled by its two cochaperones DnaJ Hsp40 and  **GrpE** .  Ref: 12654915 J Biol Chem, 2003
- The co chaperone  **GrpE**  is essential for the activities of the Hsp70  [ **DNAK** ]  system which assists protein folding.  Ref: 12840016 J Biol Chem< MedlineTA>J Biol Chem, 2003
- Deletion of  **DnaK**  s Lid Strengthens Binding to the Nucleotide Exchange Factor,  **GrpE**  A Kinetic and Thermodynamic Analysis.  Ref: 12885236 BiochemistryBiochemistry, 2003
- **GrpE**  is the nucleotide exchange factor for the Escherichia coli molecular chaperone  **DnaK** , the bacterial homologue of Hsp70  [ **DNAK** ] .  Ref: 12885238 Biochemistry, 2003
- A primer extension analysis revealed that the transcription of the  **dnaK**  operon started at the nucleotide A that is located 28 bp upstream of the  **grpE**  start codon.  Ref: 12442897 Mol Cells, 2002
- Thus, the long helix pair in the  **GrpE**  dimer acts as a thermosensor that, by decreasing its ADP ATP exchange activity, induces a shift of the  **DnaK** .substrate complexes toward the high affinity R state and in this way adapts the  **DnaK**  DnaJ  **GrpE**  system to heat shock conditions.  Ref: 12639955 J Biol Chem, 2003
- In turn, it was proposed that temperature dependent down regulation of the activity of  **GrpE**  may increase the time in which  **DnaK**  binds its substrates at higher temperatures.  Ref: 12885238 Biochemistry, 2003

**Non-interactive relationship** (e.g. studied together, co-existance, homology, etc.)

- DnaJ stimulates the hydrolysis of  **DnaK**  bound ATP, and  **GrpE**  accelerates ADP ATP exchange.  Ref: 12654915 J Biol Chem, 2003
- D Peptides as inhibitors of the  **DnaK**  DnaJ  **GrpE**  chaperone system.  Ref: 12637539 J Biol Chem, 2003
- SPR experiments revealed that  **GrpE**   **DnaK**  393 507 complex formation is inhibited by added peptide and abolished when the 33 residue flexible tail of  **GrpE**  is deleted.  Ref: 12885236 BiochemistryBiochemistry, 2003
- With disulfide stabilized  **GrpE** , the rate of ADP ATP exchange and conversion of  **DnaK**  from its ADP liganded high affinity R state to the ATP liganded low affinity T state continuously increased with increasing temperature.  Ref: 12639955 J Biol Chem, 2003
- Thermosensor Action of  **GrpE**  THE  **DnaK**  CHAPERONE SYSTEM AT HEAT SHOCK TEMPERATURES.  Ref: 12639955 J Biol Chem, 2003
- In the presence of excess nickel, NikR activated the transcription of ureA ureB hp72 73, nixA hp1077, copA2 hp1072, hpn hp1427 and hpn like hp1432 genes and repressed the expression of genes encoding proteins involved in ferric iron uptake and storage pfr hp0653, fur hp1027, frpB4 hp1512, exbB exbD hp1339 1340, ceuE hp1561, motility cheV hp616, flaA hp0601, flaB hp0115, stress responses hrcA  **grpE**   **dnaK**  hp111 110 109 and encoding outer membrane proteins omp11 hp0472, omp31 hp1469, omp32 hp1501.  Ref: 12890020 Mol Microbiol, 2003
- They provided clear evidence for a stress exerted by rice exudates, as shown by the induction of the universal stress protein gene uspA as well as genes of the heat shock regulon,  **grpE** , lon and  **dnaK** .  Ref: 12713466 Environ Microbiol, 2003
- Putative heat shock proteins  **DnaK** , DnaJ,  **GrpE** , GroES and GroEL were among the proteins whose synthesis was induced in response to acid adaptation.  Ref: 12432952 J Mol Microbiol Biotechnol, 2002
- The BIA evaluation program allowed the calculation of the equilibrium constant of the interaction of M. mazeii TBP with the promoters of the stress genes  **grpE** , hsp70  [ **DNAK** ] , and hsp40 M, respectively and, for comparison, with the promoter of a non heat shock gene, orf16 M.  Ref: 12419300 Biochem Biophys Res Commun, 2002
- It is of considerable importance for structural  **GrpE**  research to answer the question of whether the  **GrpE**  dimer is only asymmetric while bound to  **DnaK**  or also as free dimer in solution.  Ref: 12840016 J Biol Chem< MedlineTA>J Biol Chem, 2002
- Active protein disaggregation by a chaperone network composed of ClpB and  **DnaK**  DnaJ  **GrpE**  is essential for the recovery of stress induced protein aggregates in vitro and in Escherichia coli cells.  Ref: 12828638 Mol Microbiol< MedlineTA>Mol Microbiol, 2002
- However, in these conditions, the Escherichia coli  **GrpE**  dimer is asymmetric since the monomer that binds  **DnaK**  adopts an open conformation.  Ref: 12840016 J Biol Chem< MedlineTA>J Biol Chem, 2002
- Experiments were geared toward understanding the influence of  **DnaK**  s three domains, the ATPase residues 1 388, substrate binding residues 393 507, and lid residues 508 638 domains, on complex formation with  **GrpE** .  Ref: 12885236 BiochemistryBiochemistry, 2002
- A brief survey is presented of salient findings on transcription in the Archaea, focussing on stress genes of the hsp70  [ **DNAK** ] , Hsp40, and  **GrpE** .  Ref: 12540196 Crit Rev Biochem Mol Biol, 2002
- Temperature directly controls functional properties of the  **DnaK**  DnaJ  **GrpE**  chaperone system.  Ref: 12639955 J Biol Chem, 2003
- Such a function has been suggested for the  **DnaK**  system of chaperones  **DnaK** , DnaJ,  **GrpE** .  Ref: 12810912 RNA< MedlineTA>RNA, 2003
- The genes were clustered in the transcription order  **grpE**   **dnaK**  dnaJ.  Ref: 12442897 Mol Cells, 2002
- The recombinant E. coli strain simultaneously expressing the genes for CHMO, GroEL GroES and  **DnaK**  DnaJ  **GrpE**  showed a specific CHMO activity of 111 units g 1 cell protein, corresponding to a 38 fold enhancement in CHMO activity compared with the control E. coli strain expressing the CHMO gene alone.  Ref: 12827321 Appl Microbiol Biotechnol< MedlineTA>Appl Microbiol Biotechnol, 2002
- Hsp70  [ **DNAK** ]   **DnaK**  is part of the molecular chaperone machine together with Hsp40 DnaJ and  **GrpE** , and Hsp60 is a component of the chaperonin complex.  Ref: 12747562 Adv Biochem Eng Biotechnol, 2003
- For the  **DnaK**  mediated increase in the folding yield in vitro, the minimal model for APlase catalysis involves the catalyzed partitioning of a rapidly formed folding intermediate as could be inferred from the  **DnaK**  DnaJ  **GrpE**  ATP assisted refolding of GdmCl denatured luciferase.  Ref: 12553723 Biol Chem, 2002
- The low resolution structure of human  **GrpE**  presented here suggests that  **GrpE**  is a symmetric dimer when not bound to  **DnaK** .  Ref: 12840016 J Biol Chem< MedlineTA>J Biol Chem, 2002
- The association rate of the non heat shock gene orf16 was 0.4x10Ms and those for the stress genes,  **grpE** , hsp70  [ **DNAK** ] , and hsp40 were higher 2..5, and 3.5x10Ms, respectively.  Ref: 12419300 Biochem Biophys Res Commun, 2002
- Small angle X ray scattering analysis shows the protein possesses an elongated shape in solution, and demonstrates that its envelope, determined by an ab initio method, is similar to the high resolution envelope of Escherichia coli  **GrpE**  bound to  **DnaK**  obtained from single crystal X ray diffraction.  Ref: 12840016 J Biol Chem< MedlineTA>J Biol Chem, 2002
- This unique mode of binding between  **GrpE**  s tail and  **DnaK**  contributes to, but does not fully explain, the decrease in K d from 64 to 4 nM upon deletion of  **DnaK**  s lid.  Ref: 12885236 BiochemistryBiochemistry, 2002
- This induction requires both the co chaperone activity of DjlA, in cooperation with  **DnaK**  and  **GrpE** , and its unique transmembrane TM domain.  Ref: 12655402 Mol Genet Genomics, 2003
- The possibility that deletion of  **DnaK**  s lid creates a more symmetrically shaped molecule, with enhanced affinity to  **GrpE** , is also discussed.  Ref: 12885236 BiochemistryBiochemistry, 2003
- **DnaK** , a Hsp70  [ **DNAK** ]  homolog of Escherichia coli, together with its co chaperones DnaJ and  **GrpE**  protects denatured proteins from aggregation and promotes their refolding by an ATP consuming mechanism.  Ref: 12637539 J Biol Chem, 2003
- Northern blot analyses with  **grpE** ,  **dnaK** , and dnaJ specific probes revealed that the three genes are co transcribed as a 4.0 kb mRNA.  Ref: 12442897 Mol Cells, 2002
- The overall identities in the deduced amino acid sequences of  **GrpE** ,  **DnaK** , and DnaJ with those of the Escherichia coli homologs were 45. 4.5, and 61.2%, respectively.  Ref: 12442897 Mol Cells, 2002
- Here we report that d peptides efficiently inhibit the refolding of denatured luciferase by the  **DnaK**  DnaJ  **GrpE**  chaperone system EC50 = 1 2 microM.  Ref: 12637539 J Biol Chem, 2003
- In this study, we have used surface plasmon resonance SPR and isothermal microtitration calorimetry ITC to study the mechanism of complex formation between the Hsp70  [ **DNAK** ]  molecular chaperone,  **DnaK** , and its cochaperone,  **GrpE** , which is a nucleotide exchange factor.  Ref: 12885236 BiochemistryBiochemistry, 2003
- The cloned  **grpE** ,  **dnaK** , and dnaJ genes had open reading frames of 47, 926, and 16 nucleotides, coding for proteins with calculated molecular masses of 1 9 61, and 4 50, respectively.  Ref: 12442897 Mol Cells, 2002
